# Ex Vivo Treatment Response Prediction in Multiple Myeloma: Assay Formats, Clinical Correlation, and Future Directions

**DOI:** 10.3390/cancers18030411

**Published:** 2026-01-28

**Authors:** Gavin R. Oliver, Carlton C. Barnett, Kendra E. Hightower, Yubin Kang, Muhamed Baljevic

**Affiliations:** 1 Xilis, Inc., Durham, NC 27703, USA; 2 School of Medicine, University of Colorado, Denver, CO 80045, USA; 3 Department of Medicine, Duke University, Durham, NC 27708, USA; 4 Vanderbilt-Ingram Cancer Center, Vanderbilt University Medical Center, Nashville, TN 37240, USA

**Keywords:** multiple myeloma, ex vivo test, clinical correlation, review, 2D models, 3D models, dynamic systems

## Abstract

Ex vivo functional testing for multiple myeloma is rapidly evolving, yet no single assay has reached the level of reliability and clinical utility needed for routine decision-making. Existing approaches generally fall into three categories: 2D cultures, 3D models, and dynamic systems. Each contributes valuable but incomplete insight into therapeutic response. Among these, 2D assays remain the most mature, with the most extensive clinical correlations to date, though their simplified architecture limits their ability to reflect the full complexity of the bone marrow microenvironment. However, 3D systems, including spheroids and matrix-based organoids, offer improved preservation of tumor heterogeneity and microenvironmental cues. These platforms show emerging clinical relevance and may hold advantages over traditional 2D formats, and validation efforts are developing. Dynamic systems, including microfluidic models and perfused bone-marrow mimetics, represent the most physiologically ambitious category, yet their technical intricacy and early stage of development have so far limited broad clinical correlation. Altogether, the current landscape highlights substantial progress but lacks an optimal assay. In this review, we take the unique approach of examining published ex vivo tests that have demonstrated a level of clinical correlation. We evaluate their respective formats, strengths and limitations, and discuss considerations for what an ideal future assay may encompass.

## Simple Summary

This review focuses specifically on ex vivo tests for multiple myeloma drug response prediction that have demonstrated some level of clinical correlation. This creates a key distinguishing theme when compared to prior reviews in the field. By reviewing these diverse tests, their characteristics, and particularly their maturity and demonstrated clinical utility, we hope to provide a key reference manuscript that researchers and physicians alike will find invaluable as they attempt to make decisions about the design, development, or real-world clinical use of tests like these that have the potential to improve care and survival times in multiple myeloma patients.

## 1. Introduction

Multiple myeloma (MM) is a genetically heterogeneous disease caused by clonal expansion of terminally differentiated B cells, called plasma cells, primarily localized to the bone marrow (BM), that can extend through extramedullary escape or intravasation [1,2]. Although advancements in systemic therapies have resulted in markedly improved treatment responses and outcomes, with over half of patients achieving at least five years of survival [3], MM continues to represent a significant healthcare burden. There has been a gradual increase in MM disease incidence and prevalence, with MM currently representing 10% of hematological malignancies and 1% of all cancers diagnosed worldwide [4]. The annual cost of MM care in the United States exceeds USD 5 billion, with individual patient costs often reaching USD 500,000–1 million over the disease course [5]. It is the second most commonly diagnosed hematological cancer in the United States [6], and patients may present with wide-ranging organ impact manifestations—including hypercalcemia, renal injury or failure, anemia, and lytic bone lesions—typically known as CRAB myeloma defining events [7]. Quantitation of serum and/or urine monoclonal protein (M-protein) is a key component of MM diagnosis, prognosis, monitoring, and relapse detection for the vast majority of patients who harbor secretory disease, and measurement of serum free *κ* and λ light chains also plays a core role. In 2014, the International Myeloma Working Group (IMWG) expanded the diagnostic criteria for newly diagnosed multiple myeloma (NDMM) by adding three additional biomarkers of malignancy to CRAB: clonal bone marrow plasma cell percentage ≥ 60%, involved/uninvolved serum free light chain ratio ≥ 100, and >1 focal lesions (each needing to be ≥5 mm in size) on MRI studies (SLiMCRAB) [8].

Emerging biomarkers, including minimal residual disease (MRD) assessment and circulating tumor DNA (ctDNA), are beginning to complement traditional markers, offering deeper insight into treatment response and disease monitoring [9].

Treatment strategies traditionally relied on corticosteroids and melphalan, and later, combination chemotherapy [10] with autologous stem cell transplantation (ASCT) for eligible patients, but recent years have seen a transformation in treatment and responses with the introduction of modern approaches, including immunomodulatory drugs (IMiDs), proteasome inhibitors (PIs), monoclonal and bispecific antibodies (MAbs and BsAbs), antibody–drug conjugates (ADCs), and chimeric antigen receptor T-cell (CAR-T) therapies, amongst others [11,12,13,14,15,16,17,18,19]. These advances have significantly improved outcomes for MM patients, both in terms of time spent disease-free and overall survival time [20]. In fact, the new standards for frontline treatment of both ASCT-eligible and ASCT-ineligible patients, rooted in quadruplet combinations (one each of PI, IMiD, anti-CD38 mAb, and dexamethasone), are pushing the boundaries of long-term survival at previously unseen rates [21]. With the latest examination of BsAbs and CAR-T cell therapies in earlier lines of therapy, including frontline treatments, we have entered an era where “functional cure”—patients living the remaining course of their lifetime free of active disease presence—is a reasonable possibility and expectation, for at least a subset of patients. The best illustration of this reality is a recent long-term follow-up (61.3 months) update from the CARTITUDE-1 study in patients with relapsed and/or refractory MM (RRMM). This study had an expected historical median progression-free survival (PFS) of <6 months, but after a single infusion of ciltacabtagene autoleucel, one-third of patients remained treatment- and progression-free for at least five years, with no maintenance or subsequent therapy [22].

Unfortunately, despite a myriad of new treatment options, most patients experience multiple relapses, making treatment selection and toxicity avoidance paramount considerations [3,23]. Toxicity avoidance is particularly relevant as the MM incidence in elderly, frail patients with increased risk of side-effects and early discontinuation of treatment is growing [24]. The genetically heterogeneous nature of MM means that identification of robust biomarkers of drug sensitivity and resistance is challenging. Despite the emergence of improved prognostic biomarkers, including various forms of minimal residual disease (MRD) testing [25], therapeutically informative biomarkers remain lacking across all major anti-MM drug classes [26]. This makes treatment selection empiric and prediction of early treatment failures challenging. As treatment options and lines of therapy (LOT) grow, algorithms for newly diagnosed and relapsed patients are under steady revision [27,28,29,30,31,32]. In general, newly diagnosed and relapsed or refractory patients are recommended to receive combinations of therapeutic agents, and with each line of therapy, the chances of successful treatment are reduced due to declining efficacy, emerging treatment resistance mechanisms, increasing comorbidities, and accumulating toxicity [11]. Thus, despite the advances in MM treatments, there remains a critical need for providing an upfront prediction of treatment effectiveness and toxicity in patients prior to clinical planning and administration. An ideal solution would enable response prediction in a patient-specific ex vivo setting with primary patient samples routinely collected during diagnostic procedures. Multiple myeloma as a disease entity highlights the need for functional precision medicine wherein patient-derived tumor models, potentially combined with personalized ‘omics profiling, could be utilized to accurately predict treatment efficacy and toxicity prior to clinical administration of a therapeutic agent or combination therapy [33].

Creating effective models for such prediction presents significant technical and biological challenges [34]. In MM, different treatments or drug combinations induce therapeutic impact through variable mechanisms and pathways, many of which are dependent upon the components of the individual’s unique bone marrow microenvironment (BMM) [35]. This has particularly come to light with the explosion of T-cell redirecting therapies that have joined the MM armamentarium. The BM is a complex and dynamic hematopoietic organ of heterogeneous cell populations engaged in complex interplay involving both direct and chemotactic interactions [36]. This complexity, as well as widely varying disease-directed treatments, drug mechanisms, and dependencies of action, means that retention and maintenance of the BMM are considered key elements of ex vivo systems or models used to model MM biology or predict patient response to individual agents or combinations thereof.

**Figure 1:**
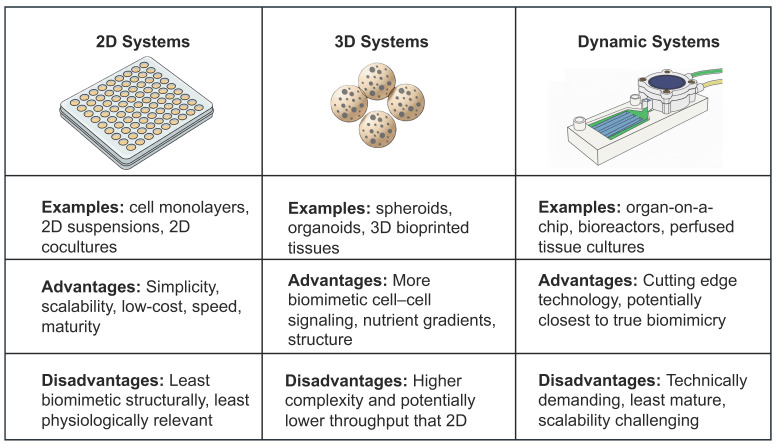
Summarized characteristics and broad strengths and weaknesses of the major classes of ex vivo systems.

Maintenance or emulation of these diverse and complex components is challenging, and wide-ranging research over the past decades has attempted to generate accurate ex vivo BM models with varying levels of success [37,38,39,40]. Models range in complexity from lower complexity cell suspension mixes [41] to more complex 3D models based on cells encapsulated in natural or synthetic hydrogels [42], and they extend to intricate microfluidic setups enabling multi-compartmental ex vivo marrow surrogate models, complete with controlled flow of microenvironment components into targeted compartments with temporal control [43] (Figure 1).

In some cases, these models have been utilized in exploratory clinical correlation studies [41,44,45,46,47,48,49,50] or clinical trials [51], with varying designs, analytical methodologies, and reported success rates. While no canonical methodology has emerged, these studies collectively highlight tangible examples that offer hope for future functionally precise medicine-informed treatment algorithms that remove empirical elements from the treatment lifecycle. Ultimately, this is the desired endpoint of any MM model—the ability to both accurately mimic in vivo biology and to create clinical benefit and improved quality of life through accurate prediction of drug response and, ideally, reduced drug toxicity and side-effects in unison.

The field of MM ex vivo modeling is one of steady and diverse advancements. This review will encompass a range of pioneering and contemporary models with the distinct prerequisite that they each report some degree of attempted correlation between assay output and a measure of patient clinical response. The assays described herein, and the research manuscripts that detail their creation and utilization, are by nature heterogeneous, meaning direct comparison is frequently challenging. For this reason, we offer a unifying definition of ‘clinical correlation’ as it applies to the criterion for inclusion in our review. In this context, clinical correlation refers to any attempt to assess and present agreement between a patient’s in-assay result and an element of their clinically described response characteristics, which is distinct from demonstrating biologically relevant aspects of an assay that are entirely detached from a patient’s treatment regimen and response.

Beyond reviewing the frequently disparate details of the individual studies, in order to maximize the utility and interpretability of this review, we aim to identify common components that enable ease of assay characterization and comparison for the reader. Unifying assay characteristics that we will consider include assay system class, sample requirements, cell populations utilized, sensitivity measures employed, measures of patient clinical response, number of patients tested, and number and type of drugs tested, as well as whether drug combinations or only single compounds were assessed. Detailed assay performance metrics, such as predictive accuracy or sensitivity, will be provided where they exist, but the extent of testing performed varies widely across studies. Assay turnaround time and clinical approval status will be discussed where they are available. These and additional granular assay details that are variably reported across studies are provided in tabular form to facilitate side-by-side comparison. By considering this compiled information, we aim to determine the strengths and limitations of the competing models and their current state of functionality, scalability, and clinical translatability. We ultimately aim to propose an optimal path forward for ex vivo MM clinical treatment response prediction. We hope this review will accelerate the field toward effective and widespread MM models with robust predictive capabilities and the ultimate ability to meaningfully improve patient care.

## 2. 2D Suspension and ‘Lower Complexity’ Systems

The BM is a semi-solid [52], 3D structure comprising solid and liquid components. It has been asserted that 2D models are insufficient for MM study or treatment response prediction due to the lack of preservation of critical components necessary to preserve the functioning of the original tissue [40]. This rationale appears to have contributed to an underrepresentation of 2D models in recent reviews of ex vivo MM models [37,39,40], as current research appears to favor the pursuit of higher complexity models that more closely represent the precise in vivo reality of the BM environment. Nonetheless, 2D models offer advantages in terms of relative simplicity, cost, throughput, reproducibility, and standardization [53,54]. Furthermore, MM is at its core a liquid malignancy that normally resides within the BM compartment [39]. Thus, despite their apparent limitations, a significant body of research has focused on models comprising basic liquid suspensions or co-cultures. Critically, these studies have frequently reported success in predicting clinical correlation.

### 2.1. EMMA (Ex Vivo Mathematical Myeloma Advisor)

Pioneering work at Moffitt Cancer Center by Silva et al. [48] led to the development of a 2D co-culture system named EMMA (Ex vivo Mathematical Myeloma Advisor), which utilized an ex vivo assay to parameterize patient drug-specific mathematical models of chemosensitivity and predict a 3-month clinical response to a panel of up to 31 drugs in a 384-well plate (theoretically up to 127 drugs in a 1536-well plate format), within 5 days of acquiring a fresh bone marrow aspirate (BMA).

MM cells were selected by CD138 expression and then co-cultured with pre-derived BM stroma and collagen in 384-well plates. Culture medium was supplemented with the patient’s own plasma and incubated overnight to allow stromal adhesion and soluble factor equilibration. Drugs were added on the second day of the assay and underwent brightfield microscopy for 4 days, with images captured once every 30 min. Digital images were algorithmically processed to quantify cell death, and dose–response curves were generated and input to parameterize the patient drug-specific mathematical chemosensitivity models. Drug-specific pharmacokinetic data from phase I studies were input to each unique patient–drug model, and 3-month clinical response predictions were generated as output within a 5-day timeframe. Optimal treatment per patient was selected by comparing profiles across all 31 single-agent drugs tested.

EMMA was tested on a cohort of 52 NDMM and RRMM patients, correctly assigning 96% of the cohort as responders or non-responders and accurately classifying 79% of patients based on more granular IMWG response criteria. EMMA was tested in two in silico clinical trials with experimental agents (venetoclax and ricolinostat) and yielded results similar to real-world phase I and II trials. This initial work suggested that EMMA may be a feasible approach to estimating drug response in MM or potentially screening for trial inclusion criteria. Since its initial publication in 2017, the test has achieved Laboratory-Developed Test (LDT) status under Clinical Laboratory Improvement Amendments (CLIA) regulations and has been run on over 600 patients, based on recently published figures [55]. Further, EMMA has been included as a component of a real-world phase II daratumumab study for older adults with NDMM [56], although results specific to the use of the assay appear to be still pending.

Assay summary: EMMA is a 2D system developed utilizing CD138+ MM cells from BMA co-cultured with BM stroma and collagen supplemented with the patients’ own plasma. Longitudinal brightfield microscopy was used to quantify cell death and construct dose–response curves that were used as input to inform patient-specific mathematical chemosensitivity models and generate predictions of 3-month clinical response based on both IMWG and binary response predictions. An optimal treatment was identified from a 31-drug panel, with a 5-day assay turnaround time. Strengths included reported high predictive accuracy in a mixed cohort of 52 NDMM and RRMM patients, CLIA LDT status, and reported real-world clinical trial use. Practical constraints included reliance on fresh tissue and single-agent-only testing.

### 2.2. My-DST (Myeloma Drug Sensitivity Testing)

Another approach, branded My-DST (Myeloma Drug Sensitivity Testing), originated at the Sherbenou lab of the University of Colorado [41]. The test was initially designed to measure treatment resistance across the disease course and has shown encouraging results as a predictive assay in both initial and follow-up studies.

My-DST was developed and tested using 55 unselected, fresh BMAs from MM cases spanning first diagnosis (24), first relapse (12), and multiple relapse (19). The initial test utilized a panel of seven drugs spanning PIs (bortezomib, carfilzomib), IMiDs (lenalidomide, pomalidomide), corticosteroids (dexamethasone), alkylating agents (cyclophosphamide), and MAbs (daratumumab). Drugs were tested as single agents ex vivo, but an in silico method (My-DST Comb) was devised to attempt prediction of combination treatment responses. My-DST testing used flow cytometry readouts to determine ex vivo sensitivity. Samples were scored as sensitive to an agent if <80% of tumor cells remained after 48 h of treatment. The study revealed that as patients underwent successive lines of therapy, their myeloma cells acquired multidrug resistance ex vivo, closely mirroring clinical observations. Drugs within a specific class showed high intercorrelation in response prediction, which was higher for IMiDs than PIs. An overall pattern observed was that drug sensitivity gradually decreased until multidrug resistance predominated after the fourth LOT. A further interesting observation was that patients experienced statistically worse event-free survival (EFS)—defined as time to progression or change in therapy due to inadequate response—if their next regimen did not contain at least two drugs to which they were predicted sensitive ex vivo by My-DST. Using My-DST, ex vivo daratumumab sensitivity was also measurable and showed a decrease after clinical exposure. The ex vivo activity of daratumumab was similar to its clinical activity and was related to its known biomarkers, i.e., ex vivo reduction of primary MM cells exposed to daratumumab correlated with the level of CD38 expression by flow cytometry, while ex vivo daratumumab reduction in primary MM cells was predominantly reversed by deactivating macrophages using clodronate-containing liposomes, suggesting that ex vivo daratumumab activity was at least partially macrophage dependent. Furthermore, daratumumab had limited effects on the viability of bone marrow plasma cells from normal donors.

My-DST Comb was devised to calculate the cumulative proportional effect of individual ex vivo drug effects using the mathematical product of the individual effects. This combinatorial effect correlated strongly with the IMWG depth of clinical response after the clinically common course of four treatment cycles (*p* = 0.0006). Using a clinical response cutoff of a 50% decrease in disease (partial response or better), a My-DST Comb cutoff of 50% was 96% sensitive (22 of 23 true positives) and 88% specific (6 of 7 true negatives). This initial study suggested clinical use potential for the My-DST assay and has been followed by wide-ranging work that continues to demonstrate the potential of the assay in treatment response prediction, novel agent development, and clinical trial use [57,58,59,60,61,62,63,64].

Assay summary: My-DST is a 2D system developed utilizing unselected cells from fresh BMAs in a mixed cohort of 55 NDMM and RRMM patients. Flow cytometry was used to quantify cell death and classify a sample as sensitive to an agent if <80% of tumor cells remained after 48 h of treatment. A panel of seven drugs was used, with agents tested individually and with combination effects inferred computationally using a mathematical method (My-DST Comb) rather than through physical drug combination. Clinical correlation for My-DST was assessed qualitatively rather than through formal predictive benchmarking. Patients were shown to experience statistically worse EFS if their next treatment regimen contained fewer than two drugs predicted as candidates by My-DST. My-DST Comb predictions showed statistically significant correlation with IMWG response criteria after four treatment cycles. Using a cutoff of partial response or better, My-DST Comb demonstrated strong ability to discriminate clinical responders from non-responders (96% sensitivity and 88% specificity). Strengths included reported high predictive accuracy of My-DST Comb in 55 patient samples and a clinically relevant turnaround time of approximately 48 h, while constraints included the lack of IMWG response correlation for My-DST and reliance on fresh tissue. Follow-up studies have reported the use of cryopreserved samples as well as other new developments that are considered in the Discussion.

### 2.3. High-Throughput Screening Approaches

Another study from Coffey et al. at the Fred Hutchinson Cancer Research Center was purportedly the first to inform MM clinical decisions in real-time [51]. The authors developed a CLIA-approved HTS assay and uniquely conducted a prospective clinical trial integrating their ex vivo assay with investigational multiomics profiling for RRMM patients. While BM or tumor biopsies were obtained from 25 patients, only 16 had sufficient tumor cells to enable HTS using the assay. CD138-selected mononuclear plasma cells from BMAs or single cell suspensions from mechanical dissociation of plasmacytomas were deposited in 384-well plates coated with a protein matrix. Cells subsequently underwent HTS with a panel of 170 approved or investigational single-agent compounds across an eight-point drug concentration range. After drug addition, samples were incubated for 72 h, and cell viability was assessed using a CellTiter-Glo (CTG) luminescent cell viability assay. Drug sensitivity was quantified using the half-maximal inhibitory concentration (IC_50_) and area under the curve (AUC) from the eight-point dose–response curves. Ultimately, a patient was defined as sensitive to a drug, i.e., considered actionable if the IC_50_ was ≤0.2 μM and that concentration was achievable safely in patients according to pharmacokinetic data. Physicians received a report highlighting the effective agents, and 13 patients were ultimately treated based on the results of the assay. Of the patients receiving assay-guided treatment, 92% achieved at least stable disease, while 46% attained partial response or better based on IMWG criteria. Each patient obtained an ex vivo global sensitivity score based on the mean area under the curve for all agents screened in the assay, and the authors demonstrated this score correlated with PFS (log-rank *p* = 0.042). Patients with more drug-sensitive tumor profiles (top 50th percentile) had longer median PFS (139 days vs. 28 days for more resistant profiles) but no statistically significant distinction in overall survival (OS). RNA and whole-exome sequencing of BM plasma cells performed on a subset of eight and seven patients, respectively, indicated that the expression levels of 105 genes and mutations in 12 genes correlated with in vitro cytotoxicity. Ultimately, the trial demonstrated that large-panel ex vivo testing was feasible with a clinically relevant turnaround time (approximately 5 days) and could impact treatment decisions in real time, although acknowledged limitations included the inability to test ~36% of patients due to low tumor yield and the small cohort size.

Assay summary: The Coffey et al. [51] assay is a 2D system that was developed and used in a prospective clinical trial with either CD138+ MM cells from fresh BMA (13 patients) or single-cell suspensions from plasmacytomas (3 patients) in a cohort of RRMM patients. A CTG assay was used to measure cell viability using IC_50_ from dose–response curves with samples considered sensitive to an agent if IC_50_ was ≤0.2 μM and safely achievable. A panel of 170 approved or investigational single-agent drugs was tested, and the agents predicted to be effective were recommended to clinicians and used to treat 13 patients based on the recommendations. The final breakdown of treated BMA versus plasmacytoma samples was not provided to our knowledge. A total of 92% of patients receiving assay-guided treatment achieved at least stable disease, while 46% attained partial response or better based on IMWG criteria. Each patient also received a global sensitivity score based on mean AUC for all screened agents, and the score correlated significantly with PFS, with the more drug-sensitive tumor profiles having longer median PFS (139 days vs. 28 days for resistant profiles). Strengths of the approach included the large HTS drug panel, clinically relevant turnaround time of approximately 5 days, CLIA approval, and use in a real-world prospective clinical trial with encouraging response rates (92% at least stable disease, 46% partial response). Limitations included fresh sample requirements, issues attaining usable tumor yields, a relatively small cohort size, and a lack of assay correlation with OS.

A Norwegian study by Giliberto et al. reported using a custom ex vivo MM test based on a range of single, doublet, and triplet treatment combinations and reported wide-ranging observational findings of potential clinical relevance [45]. The authors implemented an assay from fresh patient bone marrow samples using purified CD138+ MM cells enriched from bone marrow mononuclear cells (BMMCs) in drug-coated 384-well tissue culture (TC) treated plates. A total of 44 samples were procured at first diagnosis or relapse and processed while fresh, testing 30 single agents, 19 double-agent combinations, and 25 triple-agent combinations, which encompassed many classes of approved and investigational MM therapies. Similar to the study of Coffey et al. [51], a CTG luminescent cell viability assay was used to determine cell viability after 72 h of drug exposure, but in this study, a modified Drug Sensitivity Score (DSS) [65] on a scale of 0–100 was calculated from dose–response curves for each drug or combination. No explicit assay turnaround time was reported. Findings were wide-ranging and highly observational but frequently mirrored known clinical characteristics of MM treatment and etiology. Clinically approved combinations, such as the triplet combination of selinexor plus bortezomib plus dexamethasone, acted synergistically, and synergies required low drug concentrations to be effective. Lower response heterogeneity and higher efficacy were detected with many combinations compared to the corresponding single agents. Further, samples that were ex vivo resistant to dexamethasone generally came from patients with known steroid-refractory disease. The presence of gain (1q21) was associated with low sensitivity to venetoclax, while the presence of t(11;14) was associated with higher sensitivity. Decreased ex vivo responses to dexamethasone also reflected drug resistance observed in patients. The authors investigated synergistic drug effects using the BLISS model and reported novel synergy of melflufen plus panobinostat using low concentrations. Patients tested with triplet combinations and classified as synergistic or sensitive by their screen (*n* = 3) reached a complete response (CR) or a very good partial response (VGPR) to their current drug regimen.

In terms of actual clinical correlation, when they compared combination screening data with in vivo clinical responses, patients classified as responders (*n* = 9) tended toward significance of increased ex vivo sensitivity (median DSS of 51.8) versus patients achieving poor response, with a median DSS of 23.4 (*n* = 4; minimal response (MR) = 1, stable disease (SD) = 1, progressive disease (PD) = 2). The strength of this study lies in surveying a broad swath of therapeutics, with results delivered within approximately five days, suggesting potential for personalized decision-making. However, the cohort size was modest, findings were highly observational, and patients had limited ex vivo responses to IMiDs, suggesting a limitation of the system in its ability to retain specific, relevant cell types.

Assay summary: The Giliberto et al. [45] assay is a 2D system that was developed using CD138+ MM cells from fresh patient BM samples. The assay was evaluated in a cohort of 44 NDMM and RRMM samples. A CTG assay was used to measure cell viability, and a 0–100 scale sensitivity score was calculated from dose–response curves as input to a custom implementation of the published DSS method. The assay evaluated 74 single-, double-, or triple-agent treatments, with multi-agent treatments physically combined. Many of the study’s findings were based on observational associations with varied clinical characteristics. A small subset of three samples tested in triplet combinations and classified as synergistic or sensitive correlated with clinical outcomes, insofar as patients achieved CR or VGPR per IMWG criteria. Taken together, patients classified as responders by IMWG criteria (*n* = 9) had increased ex vivo assay sensitivity (median DSS 51.8) compared to patients with a poor response (DSS 23.4). Strengths of the approach included testing of a range of single, double, and triple agents in physical combinations and a clinically relevant turnaround time of five days. Limitations included a modest cohort size and indications that the assay failed to retain relevant cell types required for IMiD activity.

## 3. 3D Embedded Systems

While 2D models have shown significant promise in the prediction of clinical response to MM therapeutics, they lack most or all structural, morphogenetic, and microenvironmental elements of in vivo BM. A growing body of research is emerging in which efforts are made to readily and more accurately reproduce structural details of the BM cavity by embedding patients’ BM cells in 3D mixtures with varying underlying cellular and structural makeup. The theoretical advantage of 3D ex vivo models lies in their potential to more precisely reproduce the complexity and heterogeneity of the BM microenvironment. This is particularly important for MM, where the BMM plays a crucial role in disease progression, drug resistance, and treatment response. Proponents believe such approaches represent the path to increased understanding of MM disease progression, treatment resistance mechanisms, improved drug efficacy, safety, and model predictive value [40].

### 3.1. rEnd-rBM Model

Early work by Kirshner et al. [50] proposed a novel 3D BM culture model composed of two layers: a reconstructed endosteal (rEnd) layer made from collagen I and fibronectin, and an overlying central reconstructed bone marrow (rBM) matrix containing BM mononuclear cells embedded in a fibronectin/Matrigel mixture. Bone marrow samples (*n* = 48) were obtained from patients undergoing clinical biopsy, and mononuclear cells were isolated by density centrifugation. Reconstructed 3D BM cultures were generated by embedding these cells in an rBM layer and overlaying the mixture into wells pre-coated with an rEnd layer.

This combined rEnd–rBM model was demonstrated to capture important BM characteristics. The model was demonstrated to capture significant structural components of the BM and enabled the successful expansion of an MM clone. The authors asserted that this was the first molecularly verified demonstration of proliferation in vitro by ex vivo MM cells, because a 17-fold expansion of malignant cells was recorded and verified by molecular techniques to harbor the clonotypic IgH VDJ and characteristic chromosomal rearrangements. Testing of melphalan and bortezomib within the model revealed distinct targets, with melphalan targeting the hematopoietic compartment while sparing the stromal compartment. Bortezomib was shown to target only CD138 and CD56 MM plasma cells. This initial work lacked clinical correlation, but follow-up studies expanded upon the pilot, indicating clinical correlative outcomes. Several years following the initial study, a second publication describing the model appeared [66] and expanded further on the model and associated protocols. Importantly, this later work extensively profiled the survival of wide-ranging, specific native cell types and various hematological malignancies, with MM demonstrating high levels of survival and proliferation. The product became known as r-Bone and was utilized in a study predicting clinical response outcomes in a cohort of patients with MM [67]. Notably, the only record of this study is in conference abstract format, and therefore, details are less readily available than for many of the other assays discussed. The study was conducted using BMAs from 21 multiple myeloma patients. Patient BMAs were processed and established in r-Bone cultures, where they were grown for five days before being treated using the clinical regimen selected by the patient’s physician, based on the original BMA analysis. Cells were subsequently isolated from within the r-Bone platform, and both non-plasma and plasma cell population cell death were quantified using flow cytometry. The extent of cell death was correlated with observed clinical response, and r-Bone cultures were reported to correctly predict 19 of 21 MM patient outcomes, with results comprising 2 false positives, 8 true positives, and 11 true negatives. Publicly presented data suggest that correlation was to IMWG-like criteria (PD, PR, VGPR), but we have been unable to verify this or further details. The study concluded that the r-Bone system demonstrated the potential to inform treatment selection in a prospective setting.

Assay summary: The rEnd–rBM/r-Bone model is a 3D dual-layered BM culture system consisting of collagen/fibronectin endosteal layer beneath a matrix containing BMMCs in a fibronectin/Matrigel mixture. It was originally developed using 48 patient bone marrow biopsy samples and demonstrated to capture key BM characteristics. Clinical evaluation was later reported using BMAs from a cohort of 21 MM patients, with samples being treated ex vivo with a single regimen that matched the clinician’s choice of treatment based on the BMA. Plasma and non-plasma cells were isolated, and cell death was quantified with flow cytometry before the extent of cell death was correlated with an observed clinical response that appeared to resemble IMWG criteria but was not confirmed. The assay was reported to correctly predict 19 of 21 patient outcomes. Strengths of the system include a high reported level of BM biomimicry and a high degree of reported predictive accuracy when comparing assay outcomes to patient clinical responses. Limitations include the small cohort size, and clinical correlation has been described only in a conference poster and commercial promotional materials. Accordingly, many details of the clinical concordance are limited relative to other assays considered in this review.

### 3.2. Matrigel-Based Systems

Braham et al. [68] developed a Matrigel-based 3D BM model and demonstrated mesenchymal stem cell (MSC) and endothelial progenitor cell (EPC) survival and proliferation over a 28-day period. They demonstrated the ability of engineered T cells to migrate into the matrix and target and eliminate primary MM cells, demonstrating higher success in a 3D versus a 2D implementation. The same model was later used to test liposomal drug delivery (e.g., doxorubicin, bortezomib) [69] before undergoing modifications and being employed in an early retrospective clinical study published as a brief letter to the editor [47]. This Netherlands-based 3D study utilized an ex vivo growth factor-reduced Matrigel model comprising human MSCs and EPCs co-cultured with patient CD138+ myeloma cells from cryopreserved BMAs. The authors tested seven single-agent drugs spanning several major drug classes (two PIs, three IMiDs, and two alkylating agents (AAs)) at single and double doses based on known responses in 2D and 3D cultures. The study compared ex vivo response to the patient’s actual clinical response to a treatment, summarized at a drug class level, i.e., the correlation summarized across all classes, AAs plus PIs, or each class individually. Clinical correlation seems to have been assessed using a study-specific binary response classification (inferred by us due to the use of the positive predictive value (PPV) and negative predictive value (NPV)); however, explicit clinical criteria underlying these classifications were not provided. CD138+ cells were co-cultured in 3D with MSCs and EPCs for 14 days to allow the formation of primary myeloma aggregates within the cultures before treatment testing. Calcein (live) and Ethidium homodimer-1 (dead) dyes were combined to assess drug sensitivity, with resistance analyzed using flow cytometry and confocal imaging after 72 h of treatment. Responses were determined and compared with each patient’s actual clinical response to treatment by evaluating dual measurements of the percentage of dead and live cells for drug sensitivity. The authors tested two classes of correlation—strict, i.e., response to treatments received after BMA, and extended, i.e., treatments received before BMA. Predictivity was good for the strict analysis, but less so for the extended. The assay showed high predictive agreement for PIs and AAs (PPV and NPV ranging from 1.00 to 0.80 for strict, aligning well with patients’ clinical outcomes (lower for extended, ranging from 1.00 to 0.44)), but failed to predict responses to IMiDs (lenalidomide/pomalidomide), where no significant killing was observed ex vivo even for patients who responded clinically. Analyzed percentage of dead myeloma cells ex vivo, following treatment with AAs and PIs, showed the best agreement with the strict clinical treatment responses, with correspondingly high predictive values, suggesting that with the model, actual killing of myeloma cells was a superior predictor of clinical treatment responses than the live myeloma cells remaining, perhaps due to its inherent consideration of affected proliferation rates. The authors concluded that while a 3D stromal model showed success in predicting response to select drug classes, it was not an all-encompassing solution, and that immune components were likely required to be present in order to successfully profile all therapy classes.

Assay summary: The Braham et al. [47] assay is a 3D Matrigel-based model. It utilized human MSCs and EPCs co-cultured with patient CD138+ MM cells isolated from MNCs derived from cryopreserved patient BMAs. The assay was evaluated in a cohort of seven RRMM patients. Flow cytometry and confocal imaging with live and dead dyes were used to assess sample response to treatment, utilizing a percentage of live and dead cell counts. Assay responses were compared to patients’ clinical response to treatment, although a precise response metric formulation was not described. We infer that clinical response was based on a binary responder/non-responder split due to the study’s use of PPV and NPV values. Response to treatments received before and after BMA was assessed. PPV and NPV ranged from 1.00 to 0.80 for PIs and AAs when assessing treatments received post-BMA but were lower for treatments received pre-BMA (1.00–0.44). Higher predictive accuracy was obtained from dead cell counts than live cell counts. The assay failed to predict responses to IMiDs due to a lack of cell killing observed in the assay that was independent of patient clinical response. The assay evaluated seven single agents. Strengths of the approach included reported high PPV and NPV for AAs and PIs using dead-cell counts, as well as the ability to utilize cryopreserved samples. Limitations included a small cohort size, brevity of the manuscript, ambiguity in certain study elements, the relatively small drug panel, and the stated inability to utilize IMiDs.

### 3.3. 3DTEBM^®^ Model

A study by Alhallak et al. [46] built upon earlier work by de la Puente et al. [70] to assess clinical correlation using a 3D tissue-engineered BM (3DTEBM^®^) model utilizing a mixture of patient-derived myeloma, plasma, endothelial, and stromal cells from BMA to test drug regimens ex vivo, without the addition of exogenous materials. The authors used autologous BM supernatant to create the matrix, whereby a hydrogel-like structure was created by crosslinking endogenous fibrinogen, resulting in a culture said to be softer and more biomimetic than synthetic polymers. Fresh versus cryopreserved BMA was not explicitly stated; however, the use of autologous BM supernatant and the biomimetic nature of the model suggest fresh processing. The authors also stated that all growth factors, enzymes, and cytokines naturally found in the TME were included, thus better recapitulating the BM niche found in vivo. A cohort of 19 RRMM patients was enrolled in the study, and each patient’s upcoming treatment regimen was applied to their 3D BM culture, and subsequently, CD38+ live MM cells were quantified by flow cytometry. Treatments included single-agent and two- or three-drug combinations of 11 agents, spanning multiple drug classes, testing physical drug combinations ex vivo rather than relying on computationally predictive methods. The full list of drugs tested was carfilzomib, bortezomib, ixazomib, panobinostat, lenalidomide, pomalidomide, dexamethasone, etoposide, doxorubicin, daratumumab, and melphalan. Steady state plasma drug concentration (Css) was determined based on pharmacokinetic data obtained from phase 1 and/or phase 2 clinical trials, and untreated samples, 3× Css, 10× Css were input to a single-factor ANOVA. Samples were defined as responsive or unresponsive based on a statistically significant loss of viability (*p* < 0.05 by ANOVA) and compared with untreated controls with parallel assessment by MTT assay. The IMWG criteria-based response to the same regimen was used in testing clinical correlation. Patients achieving partial response (PR) or better were considered “responsive,” while those with SD or PD were “non-responsive,” and ex vivo results (sensitive vs. not) were compared to this outcome. Predictions agreed with clinical outcomes in 89% of cases. The study correctly identified 100% of non-responders and 75% of responders. Traditional 2D culture assays were also tested, and the authors reported no correlation with patient clinical response, concluding that this underscored the importance of the 3D BM microenvironment in ex vivo testing and that 3DTEBM^®^ represents a promising approach for studying primary BM malignancies.

Assay summary: The 3DTEBM^®^ model is a 3D system developed using unselected patient-derived myeloma, plasma, endothelial, and stromal cells from BMAs (likely fresh based on inference), using a hydrogel-like structure created using endogenous fibrinogen. All growth factors, enzymes, and cytokines naturally found in the TME were stated to be included in the model. The model was evaluated in a cohort of 19 RRMM patients. Patient samples were treated with their upcoming clinical regimen, which spanned 11 agents in single-drug format, or two- or three-drug combinations ex vivo. Untreated samples as well as samples treated with drugs at concentrations of 3× Css and 10× Css were analyzed using flow cytometry to quantify CD38+ live MM cells, and results were input to a single-factor ANOVA. An ANOVA result showing loss of viability with *p* < 0.05 was used to define response, compared with untreated controls, with parallel assessment using the MTT assay. IMWG-based clinical response to the same regimen tested in the assay was used to determine clinical correlation. Patients with PR or better were considered responsive, while SD or PD was classified as non-responsive. Ex vivo binary sensitivity results were compared to this outcome. The study correctly identified 100% of non-responders and 75% of responders, with overall agreement of 89%. Strengths of the approach included an apparently highly biomimetic model and good overall accuracy in predicting a patient’s response to an actual clinical treatment, including up to three drug combinations, in a heterogeneously treated cohort. Limitations included a likely requirement for fresh patient samples and a relatively small patient cohort.

### 3.4. PuraMatrix™ System

A study by Jakubikova et al. [42] utilized a synthetic self-assembling hydrogel (PuraMatrix™) to generate a novel model designed to mimic the neoplastic BM microenvironment, with cultures being maintained for up to 3 weeks. The 3D system was established by co-culturing primary MM patient BM cells from fresh BMAs with MSCs in the hydrogel. The authors compared a 2D model in parallel and asserted that the 3D model more closely mimicked MM marrow physiology. Increased expression of wide-ranging chemokines and extracellular matrix (ECM) components was reported, which the authors stated provided a better model to explore MM and suggested might better assess tumor sensitivity to anti-MM therapies and inform single-agent or combination therapy. To study MSCs at different stages of MM, the authors used a multicolor flow cytometry panel. The percentage of MSC population was compared in smoldering MM, NDMM, RRMM, and relapsed MM. In one 10-patient cohort, patient-derived MSCs were cultured in 3D and 2D models for 5 days, prior to the addition of anti-MM drugs for 3 days. The 3D co-culture appeared to confer greater resistance than 2D for several agents. Specifically, 3D-cultured myeloma cells survived better with drugs such as lenalidomide, thalidomide, and bortezomib, compared to 2D, whereas carfilzomib still induced high cell death even in 3D. This 10-patient MSC group was said to demonstrate the general effect of a 3D BM environment conferring drug resistance, consistent with what is observed in patients with “tough-to-treat” myeloma.

Attempts to correlate assay results to known clinical outcomes were only a minor component of this study. In a second cohort of four individual patients with no explicitly reported disease stage but known resistance to pomalidomide or carfilzomib, BM samples were tested using both the 3D and 2D models in parallel. Specifically, freshly isolated mononuclear cells (MNCs) containing each patient’s myeloma cells were co-cultured with MSCs and treated with pomalidomide or carfilzomib for 7 days. The authors then determined how each patient’s myeloma cells responded in each model and compared the fold change to the untreated condition. Resistance to pomalidomide was observed in two patients and resistance to carfilzomib in one patient in the 3D model but not in the 2D model, reflecting the patients’ known clinical resistance to the respective agents. Patient-level correlative data in the study was limited in its extent, however, and the authors concluded that a higher number of patients were needed to validate their results, stating that prospective trials were underway to assess the model’s value in predicting treatment response in MM.

Assay summary: The PuraMatrix™ system is a 3D hydrogel-based model that was developed using primary MM patient-derived bone marrow cells co-cultured with MSCs obtained from fresh BMAs. Limited clinical correlation was explored in a cohort of four MM patients with known clinical resistance to pomalidomide or carfilzomib but no formal classification of disease stage (NDMM vs. RRMM). Freshly isolated mononuclear cells containing each patient’s myeloma cells were embedded in the 3D matrix and treated ex vivo with either pomalidomide or carfilzomib. Treatment response was assessed by comparing the fold change in MM cell survival relative to untreated controls. Clinical relevance was evaluated qualitatively by comparing ex vivo resistance patterns to patients’ known clinical responses to the same agents. Resistance to pomalidomide was observed ex vivo in two patients, and resistance to carfilzomib was observed in one patient, consistent with their documented clinical resistance. Clinical correlation was limited to observational, per-patient concordance rather than formal benchmarking. Strengths included the use of a biomimetic 3D microenvironment and physical testing of patient-specific therapies, while limitations included the very small cohort size, small number of agents tested, and the exploratory nature of the clinical correlation analysis.

## 4. Dynamic Systems

Increasingly complex systems are being explored in the pursuit of an ideal BM model. Such approaches include methods like organ-on-a-chip, other intricate microfluidic devices, or wholly distinct novel complex technologies [40,71]. These higher-complexity models can enable the assessment of complex characteristics that may be difficult or impossible to include in more traditional 2D or 3D culture systems, such as the active circulation of waste or nutritional components. Such models may represent a path toward a truly biomimetic solution, but they currently face challenges in terms of their overall maturity. Many have been described in detail in prior works [37,39,40,71] but largely fall outside the scope of this review due to the critical lack of attempted clinical correlation. Despite the challenges involved, isolated reports of studies attempting sometimes very limited clinical correlation analysis utilizing these modern paradigms have been published and are considered below.

### 4.1. RCCS™ Bioreactor System

Ferrarini et al. utilized a dynamic 3D model in the form of the RCCS™ Bioreactor [72] and reported encouraging initial results in what was described as a proof-of-principle pre-clinical study. This model was based on the application of microgravity technology to ex vivo specimens in a horizontally rotating bioreactor whose culture vessel is fluid-filled to eliminate headspace between the culture medium and atmosphere. Operational conditions were monitored throughout an experiment, optimizing for laminar flow of fluid within the culture chamber while 3D tissue samples remained in a condition of ‘free-fall’ that minimizes shear forces associated with impeller-stirred bioreactors. These conditions also minimize tissue sedimentation, while the vessel shape favors optimal oxygenation.

The authors reported successful ‘long-term’ (~2 week) culture of myeloma tissue explants with viability of myeloma cells within a native microenvironment that included bone lamellae and vessels, as confirmed by histological examination. Matrix Metalloprotease activity, as well as VEGF, Angiopoietin-2, and β2 microglobulin levels, were evaluated in 3D-culture supernatant and reflected the effects of bortezomib treatment. Levels of β2 microglobulin following bortezomib-based therapy from supernatant derived from samples and patients’ sera showed positive concordance with drug response, both ex vivo and in vivo. In select cases, isolated plasma cells were evaluated by flow cytometry to support evaluation of sensitivity to bortezomib in tissue explants. Parallel experiments used transmission electron microscopy (TEM) to confirm ultrastructural features of bortezomib-induced cell death, including nuclear condensation and cytoplasmic vacuolization, with the authors ultimately reporting that ex vivo exposure to bortezomib produced cellular effects that were consistent with known MM biology.

Despite the technology’s novel functionalities, only very limited observational clinical correlation has been reported. Clinical correlation was reported using tissue samples from two patients with known differences in bortezomib sensitivity. One patient had newly diagnosed MM and was responsive to bortezomib, with tissue obtained from a skull lesion prior to therapy. The other patient had failed multiple prior lines of therapy and was known to be clinically refractory to bortezomib at the time of sampling. Tissue for this patient was obtained from a subcutaneous abdominal lesion biopsy. Both patients were reported as DSS stage IIIA, while ISS stage III was reported for the newly diagnosed patient but unknown for the other. Tissue explants from each patient were cultured in the RCCS™ bioreactor and treated with bortezomib for seven days.

Treatment effects were qualitatively evaluated using histological and immunohistochemical examination of explants, focusing on the presence or disappearance of plasma cells within the native tissue architecture. In the patient who was responsive to bortezomib, the ex vivo assay produced the disappearance of plasma cells in tissue explants consistent with the patient’s clinical response. Bortezomib treatment did not reduce plasma cell burden in explants from the second patient, in agreement with the known lack of clinical response. Notably, bortezomib treatment reduced MM-associated microvasculature in explants from both patients, irrespective of differential cytotoxic effects on plasma cells, suggesting distinct drug effects on tumor versus microenvironmental compartments.

While these observations were qualitative and limited to two patients, the authors concluded that the RCCS™ bioreactor system was capable of replicating clinical patient drug responses in intact MM tissue.

Assay summary: The RCCS™ bioreactor is a dynamic 3D culture system used to maintain intact myeloma tissue explants under low-shear conditions. Proof-of-principle clinical correlation was assessed in two patients with known clinical response to bortezomib. Fresh tissue explants, including native stromal and vascular components, were cultured and treated with single-agent bortezomib. Tissue was obtained from a skull and a subcutaneous lesion in the patients, respectively. Treatment effects were assessed using histological and immunohistochemical examination of plasma cells within the native tissue architecture. One newly diagnosed bortezomib-sensitive patient and one patient who had failed multiple treatment lines and was clinically bortezomib resistant at sampling were assessed using the assay. Both patients were reported as DSS IIIA, while ISS was stage III for the bortezomib-sensitive patient and unknown for the other patient. Ex vivo findings were in agreement with the clinical responses by qualitative assessment. Strengths included an intricate model with preservation of tissue architecture and microenvironmental elements, and an inferred clinically relevant turnaround time since explants were cultured with drugs for seven days. Limitations included the very small number of cases used in clinical correlation, the qualitative assessment of concordance, and the use of only a single drug.

### 4.2. Microfluidic Approaches

Pak et al. [49] utilized a custom microfluidics-based approach [73] in which they successfully correlated ex vivo bortezomib response with clinical patient response data. This approach was based on a platform previously published by the same group, which had enabled successful culture and functional analysis of small numbers of suspended cells in co-culture with alternative cell species placed in separate compartments and shown to successfully communicate via soluble factors through diffusion ports [73]. While this approach enabled analysis of soluble interactions between MM and non-tumor companion cells, it did not account for effects mediated by direct contacts between MM cells and non-MM cells. In this follow-up study [49], CD138+ tumor cells were sorted within one day of bone marrow aspiration and cultured in either mono- (MicroMC) or cis-co-culture [MicroC(3)] with the patients’ own CD138− non-tumor MNC fractions. Primary CD138+ cells were exposed to bortezomib in MicroC(3), and the patients’ own CD138− companion mononuclear cells were included to capture a subset of the patient-specific tumor microenvironment within the platform. Artificially magnified contributions of a specific non-tumor cell type were avoided, and preservation of relative contributions of other non-tumor cell types was attained by including the entire mixture of CD138− cells in the side chambers of the MicroC(3) assay. In sum, this cis-co-culture approach was considered novel as prior approaches had utilized trans-culture or monoculture techniques. The novelty of co-culturing with same-patient normal cells and the rapid turnaround time (three days) offered the possibility of a more biomimetic and clinically valid assay. MicroC(3) was tested by measuring the ex vivo bortezomib toxicity responses of MM tumor cells in MicroC(3) versus a comparison group cultured in microfluidic monoculture lacking co-cultured non-tumor cells (MicroMC). Fluorescence microscopy with live and dead fluorescent antibodies (calcein AM and ethidium homodimer) was utilized to measure treatment effects. Gaussian mixture model clustering indicated that ex vivo patient MM cell responses from 11 of 15 patients correlated with respective patient IMWG clinical responses in MicroMC, while 17 of 17 patients matched their respective clinical responses using MicroC(3). Therefore, MicroC(3) successfully identified all clinical responses for patients exposed to therapies containing bortezomib. Of further note was that the MicroC(3) clusters separated into sensitive and non-sensitive groupings by k-means and Gaussian mixture clustering, regardless of whether the BMA was acquired prior to or after bortezomib-containing therapy, unlike other studies, which had reported differential abilities to predict responses between these sampling timepoints.

Assay summary: The MicroC(3) assay is a dynamic microfluidics-based system that was developed using CD138+ MM cells sorted within a day of BMA collection and co-cultured with patients’ own CD138− MNCs. The assay was evaluated in a cohort of 17 patients described by the authors as newly diagnosed, sensitive, relapsed, refractory, or relapsed/refractory. Approximately equal proportions of the cohort had their BMA collected pre-treatment or alternatively post-treatment. Treatment effects were measured by exposing patient primary cells to bortezomib and using fluorescence microscopy with live and dead markers. Gaussian mixture model clustering correctly predicted patient IMWG clinical response category from ex vivo responses in all patients tested. Gaussian mixture and k-means clustering correctly grouped patients into binary responder/non-responder clusters regardless of whether BMA was collected pre- or post-treatment. Strengths of the assay included correct prediction of IMWG category for every patient, a clinically relevant three-day turnaround time, evidence of binary discriminative ability regardless of BMA time of collection relative to treatment, and evidence that inclusion of CD138− MNCs improved assay performance. Limitations included the modest cohort size and the testing of only a single agent across all patients.

### 4.3. Advanced Imaging Systems

Another study from a German research group utilized a novel microwell-based ex vivo assay for measuring drug responses in primary MM cells and recording corresponding immune cell activity. Testing occurred using a CellPly Vivacyte with a CC-Array microfluidic device containing 16 channels, each with 1200 microwells, which could be analyzed individually. One condition per channel could be measured simultaneously, and all preparation steps, such as cell staining, washing, and suspension in different concentrations of the drugs, could be automated by the instrument. The system employs image-based analysis through fluorescence detection of tumor and effector cells and can detect cell co-localization and cell–cell contact. In this study by Krüger et al. [44], BM mononuclear cells were obtained from 12 NDMM and 10 RRMM patients from EDTA BMAs processed on the day of collection. A panel of six drugs (bortezomib, melphalan, dexamethasone, lenalidomide, daratumumab, and elotuzumab) was tested, and subsequently, CD138+ cells were quantified by live/dead staining. In this setup, ≤90% viable tumor cells (≥10% kill) were considered responders, whereas >90% viability was classified as indicative of non-response to the drug. Daratumumab, elotuzumab, and lenalidomide ex vivo responses largely did not correlate with the clinical courses of the patients, and responses were only achieved with high drug concentrations. The authors asserted this was likely due to the short assay time. Meanwhile, the authors reported that the assay platform detected mostly concordant effects of bortezomib, melphalan, and dexamethasone ex vivo vs. in vivo, although notably a range of different correlative measures was used, testing correlation with previous treatments, subsequent clinical responses to the same drug, responses from drugs in the same functional drug class or to a treatment combination containing the drug. MM cell viability of each patient for each substance tested was compared with LOT, Revised Multiple Myeloma International Staging System (R-ISS), and pretreatment with the substance class. The authors asserted that a direct correlation between the individual in vivo response to each drug was impossible since all patients received combination treatments and the drugs administered in vivo frequently did not match the drugs tested ex vivo. A response score was defined for the correlations using the lowest cell viability for each substance independently of time point or concentration, a measure described as enabling compensation for the variability of cell viabilities between patients. Correlation between the in vitro drug response and the number of therapy lines was evident for some patients, but with no statistically significant difference observed. There was also a trend to higher rates of in vitro resistance in patients with multiple relapses. A high in vitro response (MM viability < 70%) across all tested substances was seen in 68.4% of cases among patients without any previous treatment, in 62.5% of cases with LOT of 1–2, and in 14.3% of patients with LOT > 2. The highest proportion (42.9%) of in vitro resistance (MM viability > 90%) was found in patients with >2 LOT, while there was no case of resistance among treatment-naïve patients. A notable and promising aspect of this platform was the demonstrated ability to measure natural killer- and T-cell-mediated cytotoxicity using image-based statistical analysis of cell proximity and killing events within wells. This degree of visual and analytical definition offers insight into a potentially routine ability of future-state assays, with immune-mediated killing being directly observable versus assumed based on administered drug class, or upon conclusions drawn from limited immune component depletion experiments subsequent to orthogonal cell viability assessments.

Assay summary: The Krüger et al. [44] assay is a dynamic microwell-based system based on the Cellply Vivacyte system with a CC-Array microfluidic device and developed using unselected MNCs from fresh EDTA BMAs. The system was evaluated in a cohort of 12 NDMM and 10 RRMM patients. Assay readout was based on live/dead staining of CD138+ cells and microwell-based fluorescence imaging. A panel of six drugs, including PIs, AAs, mAbs, IMiDs, and corticosteroids, was tested. The clinical correlation components of this study were multi-metric, heterogeneous, and indirect in nature. Ex vivo drug responses were compared to multiple clinically relevant measures, including prior treatment history, subsequent clinical responses to the same or related agents, treatment combinations containing the tested drug, and disease burden metrics, including LOT and R-ISS. A response score based on the lowest MM cell viability observed across concentrations and time points was used to enable comparison between patients. Using this framework, concordance between ex vivo and clinical response was most evident for bortezomib, melphalan, and dexamethasone. Responses to mAbs and IMiDs had limited clinical agreement. Overall, the analysis supported qualitative and class-level clinical relevance rather than formal predictive benchmarking. Strengths included evidence of correlation with clinical features across multiple drug classes, the ability to assess immune cell proximity to MM cells, and the reported ability to perform drug-sensitivity analysis over a duration of ~2 days for most patient samples. Limitations included a small cohort size and the lack of correlation with direct quantitative clinical measures.

## 5. Discussion

### 5.1. Patterns and Trends Across Assay Classes

Our consideration of the literature reveals a diverse body of research whose heterogeneity extends beyond our fundamental classification of assays as 2D, 3D, or dynamic. Within this initial level of classification, there exists a spectrum of nuanced contrasts between studies, encompassing characteristics such as the technology used to measure cell viability, the assay vessel, the mechanisms and cutoffs used to define sensitivity or resistance, clinical endpoints measured, the methods used to define clinical correlation, the clinical makeup of the cohort, its size, and multiple other key characteristics. Not unexpectedly, the number of patients assessed for clinical correlation and the degree of quantitative rigor vary widely across studies and frequently reflect disparities in assay maturity or complexity. These details are described within the main text and are captured and unified by Table 1A–C in a more structured and granular format to facilitate fine-grained comparison of 2D, 3D, and dynamic models side-by-side [...].

To briefly illustrate, consider the EMMA [48] and My-DST [41] tests, which represent two of the more mature assays described. While both are classified as 2D assays and each has been widely utilized either clinically or in follow-up research and pharmaceutical collaboration, they are very different assays. EMMA uses brightfield microscopy and proprietary algorithms to determine drug sensitivity, while My-DST utilizes flow cytometry and a hard numerical cutoff for percentage of cell survival. The EMMA study investigated 31 drugs while My-DST originally assessed 7. While both report clinical correlation, My-DST reports a higher correlation than EMMA using IMWG criteria, but both assays use entirely different mechanisms, calculations, and cohorts to determine their correlation scores. Meanwhile, My-DST also reports correlation with EFS, while EMMA reports results on the basis of IMWG criteria alone. Notably, these represent two of the larger cohort studies described in this review. In some of the studies described, clinical comparisons are based on as few as two patients, and real-world value becomes difficult to extrapolate. Evidence that a therapeutic agent’s mechanism of action within an assay is similar or identical to its in vivo activity is lacking, and in the case of IMiDs especially, is often assumed based on the degree of cancer cell killing, rather than being borne out through exhaustive profiling of immune components to demonstrate they are present, observed, and functionally involved. Therefore, it is difficult to directly compare assays even when clinical correlation is reported, and it is equally difficult to select a ‘best’ assay from those described. Nonetheless, it seems possible to consider the assays described as a cohort and to summarize the overall maturity of each class and the most prominent assays’ degree of proven utility, before considering which characteristics might be most desirable in an assay built on the learnings of this review.

Currently, 2D tests appear to occupy a dominant position within the space. Despite the shortcomings described in many original research articles and reviews [37,39,40] as well as multiple reports within this review of 2D models falling short in head-to-head testing with 3D models [42,46,47], they constitute the class with the most mature tests, the widest clinical reach, and the most published utility in drug development. The number of drugs per assay in the 2D studies also frequently and markedly exceeds that from the other assay classes. Further, the clinical correlation reported by the 2D studies, while subject to critical interpretation, is frequently compelling. Both EMMA and the assay of Coffey et al. [51] are CLIA-approved and actively being run on patient samples, while My-DST is understood to be in the process of clinical validation. The team behind EMMA released a request for industry and academic partners to expand their test rollout several years ago, reporting over 600 patients profiled at that time [55], and we can assume this number continues to rise. My-DST has been used in the investigation of multiple novel treatments and in clinical studies. Follow-up work has included studies of isatuximab [57], omacetaxine [60], elranatamab after BCMA CAR-T [59], the CD38 × ICAM-1 bispecific antibody VP301 [58], the anti-CD38 T-cell engager SAR442257 [61], MYCi975 [62], treatment improvement in frail patients [63], and in a phase II trial [64].

Despite the apparent favor of 2D tests, as stated at several junctures within this review, evidence exists suggesting the necessity of a 3D scaffold alongside BMM components to accurately represent the MM disease niche for therapeutic effect prediction. Moreover, the body of research supporting such assertions is growing. Several 3D studies report positive clinical correlation results. Cohort sizes studied within this category frequently match or approach the size of the 2D studies, particularly the work of Jakubikova et al. [42] as well as the clinical correlation studies using 3DTEBM^®^ [46] and r-Bone [67]. It is also worth reiterating that several of the 3D studies reviewed here compared 2D and 3D models directly, identifying inferior side-by-side performance of the 2D models [42,46,47]. Several of these 3D works have produced ongoing success of varying degrees, with r-Bone being marketed commercially. It is stated that this platform is an optimal system for small molecules, mAbs, antibodies, checkpoint inhibitors, ADCs, bispecific T-cell engagers (BiTEs), and CAR-T cells. It is unknown whether clinical studies using r-Bone continue to be conducted today by the original study authors or others. While the study of Jakubikova et al. [42] stated that prospective trials were ongoing at the time of publication, this work has not appeared in any peer-reviewed format.

In general, the publications describing dynamic systems are of a much more limited scope. Since the original publications detailing the dynamic and microfluidic-based models reviewed herein, there have been relatively few new developments broadening their assay use and availability. The MicroC(3) platform described by Pak et al. [49] did transition toward commercialization when the authors formed a company and SBIR funding was awarded to perform a 20-patient clinical trial and develop the test as a companion diagnostic [74]. The work of Ferrarini et al. [72] and Kruger et al. [44] were each built upon commercially available platforms that continue to be marketed, but the studies themselves do not appear to have yielded visible follow-up work or available assays. It is our opinion that these emerging, often complex technologies capture important biomimetic characteristics that may represent a future state of testing but currently remain too immature or intractable for medium- to high-throughput, patient-focused studies. The dearth of even modestly scaled clinical correlation studies utilizing these systems in the published literature, and the lack of meaningful follow-up work post-publication, is likely reflective of this fact.

### 5.2. Comparative Performance of Assay Classes

In summary of the above, 2D assays appear to be the most mature class and have undergone the most extensive validation in terms of patient numbers per study (median 32.5) and drug numbers or combinations tested (median 52.5). All studies included some attempt at correlation testing with commonly accepted clinical measures like IMWG criteria. Furthermore, 2D assays are the class most likely to have been included in real-world clinical trials and are the only class to contain a prospective clinical trial or CLIA-approved assays. Accuracy is difficult to compare across classes due to the disparities in methods of assessment. However, EMMA reported 96% of patients correctly classified by binary response prediction and 79% accuracy using IMWG criteria, while myDST Comb reported 96% sensitivity and 88% specificity using binary response criteria and a strong correlation (*p* = 0.0006) with IMWG criteria after four treatment cycles. The Coffey et al. [51] assay reported 92% of patients treated based on assay predictions achieving at least SD, and 46% of patients achieving PR or better. The work of Giliberto et al. [45] reported results that showed a correlation between assay and patient results but was much more observational and less quantitative. Turnaround times are often more explicitly stated for 2D assays and are clinically relevant in terms of being typically five days or fewer.

In contrast, 3D assays frequently have the benefit of reported higher biomimicry but generally smaller numbers of patients (median 13). Like the 2D tests, 3D studies frequently employed commonly accepted clinical measures, such as IMWG criteria, but 3D studies also generally considered fewer drugs and drug combinations (median 9). 3D models have widely varying methods of assessing clinical correlation. However, the r-Bone study reported 90% accuracy with what appeared to be IMWG-like criteria, although this was not directly specified. Alhallak et al. [46] reported predictions concordant with clinical outcome in 89% of cases based on IMWG criteria. Braham et al. [47] reported high predictive agreement (PPV 1.00–0.80) for response to treatment received after BMA, while Jakubikova et al. [42] performed limited, observational per-patient concordance using their PuraMatrix™ system. The 3D models were less likely to explicitly list a turnaround time, although most appeared clinically operable based on reported culture and drug treatment durations.

Dynamic models benefit from the highest intricacy of experimental setup and evidence of being the most biomimetic class of assay. However, the number of patients tested in clinical correlation was frequently lacking (median five), with one study only assessing two patients against clinical correlates. They are also the class of assay with the least expansive drug testing and clinically correlative work, with two studies assessing only patient sample sensitivity to single-agent bortezomib and a third testing only single-agent dexamethasone. Dynamic models, whilst not explicit in speaking of clinical turnaround times, generally appear to yield clinically compatible turnaround times based on assay durations.

### 5.3. Heterogeneity and the Potential for Standardization

It is important to revisit and emphasize the previously described issue of heterogeneity across the studies reviewed herein. Factors such as the clinical parameters utilized in correlative efforts (e.g., PFS, IMWG, LOT), and the statistical methodologies employed are disparate and frequently cause inter-assay comparisons to be challenging, as well as creating clear difficulties for a reader to make a firm judgement of an assay’s true clinical utility. Part of this challenge undoubtedly stems from the fact that all the studies considered originated in medical or academic research laboratories, where mindsets may differ, and the dual and sometimes conflicting priorities of publication versus improvement in patient care can lead to bifurcations in focus and practice. For this reason, an absolute standardization of development efforts and reporting is unlikely in our opinion. Nonetheless, a coordinated movement toward homogenization within the MM community would indeed be a worthwhile pursuit. The European Hematology Association (EHA) is involved in a ‘standards for functional precision medicine’ project [75], with one of their stated goals being the comparison of screening platforms and readouts; however, to our knowledge, no formal guidelines have yet been released. Particularly in the light of current ongoing initiatives to migrate previously animal-based research toward New Approach Methodologies (NAMs) by organizations such as the NIH [76] and FDA [77], there are emerging opportunities for improved, standardized recommendations and requirements at both the disease-agnostic and disease-specific level, and we could foresee and would recommend collaborative work in the areas of standardization among bodies such as these and organizations like the EHA and their international counterparts. Similarly, coordination with industry partners in the biotechnology and pharmaceutical fields could further facilitate wider change. Certainly, we see great value in these efforts and are hopeful that the future of nascent efforts such as those described above will be drivers of positive change.

### 5.4. Toward an Optimal Ex Vivo MM Assay

With all this considered, we again turn to the question of what might represent an optimal assay. While the future holds significant promise in terms of novel and potentially intricate next-generation technology building upon the cutting-edge technologies of today [44,49,72], we will aim to focus our opinion on the nearer term rather than the distant future. Thus, an ideal assay should be achievable in the immediate to near term without requiring many years of further research and development. For any idealized assay to win the trust of an end-user, clinical correlation data is required. Without this, it becomes impossible to elicit faith in a test’s real-world performance. Furthermore, correlative data should be statistically robust, peer-reviewed, and generated on the largest cohort reasonably possible, with a diversity of disease states captured. Ideally, clinical endpoints should be based on a well-established, short-term response framework, like the IMWG criteria. The use of survival endpoints such as EFS or OS may be of interest to subsets of researchers or clinicians and might optionally be explored; however, these are problematic since they are confounded by factors including LOT and difficulty in collecting and correlating such data, and are therefore infrequently employed [78,79]. The use of newer emerging measures, such as MRD and clonal evolution, has much to offer the field and is likely to assume a larger role in future clinical correlation studies like those described here. While many studies in our review have consistently utilized IMWG, others have been more ambiguous in their measurements, and none have explicitly applied modern alternatives. It is our belief that greater standardization should occur.

The existence of supporting data from prospective studies, as opposed to purely retrospective, would be welcome, but of the works considered here, only the work of Coffey et al. [51] achieved that, and we must accept the logistical difficulties associated with routinely achieving this. An assay should ideally offer utility in both the clinical and research settings to serve the various industries whose synergy ultimately leads to improved patient care. Thus, the assay would possess features relevant to clinicians and to their counterparts within pharmaceutical or biotech entities. To be broadly applicable, the test must have been successfully tested on an appropriately representative catalog of therapeutic agents to be either clinically informative at a given stage of treatment or to have proven the ability to be mechanistically compatible with agents of interest to the entities involved in the development of novel therapeutics. Clinical use cases likely necessitate the ability to profile a wide array of agents simultaneously, and most frequently in combination formats, which might be achieved through in silico or ex vivo mechanisms. It is unlikely that any current assay will capture every available agent and combination thereof, so ideally, this would be compensated for through an assay’s demonstrated flexibility in adapting to new agents as they enter development or clinical care regimens. Drug development applications may be more tolerant of test formats that are mechanistically accurate without the same requirement for myriad testing in parallel.

It is necessary that an assay should have a turnaround time that is conducive to both influencing clinical care and enabling the timely turnaround of associated research. This can be facilitated by the assay implementing significant levels of automation, which will also contribute to the key characteristics of consistency and reproducibility fundamental to any successful assay. Tissue or cell input requirements must be achievable within the confines of standard clinical practice, since most tests will be dependent upon clinically procured samples, primarily BMAs of limited volume with complex handling requirements.

Immune-mediated effects play a major role in multiple MM treatment paradigms, particularly in less advanced stages of the disease that precede immune exhaustion. Tests intended to capture immune-mediated treatment effects will ideally incorporate robust quantification of relevant immune and microenvironmental components, including presence, functional state, and impact on tumor burden. While it is unlikely that most assays will capture 100% of the BMM stably and reliably, despite the work of Kirshner et al. [50,66,67] claiming to come close, there should be compelling evidence of preserved BMM components relevant to the activity of the therapeutic agents being profiled, and this evidence should span the runtime of the assay, ensuring that components of interest are present and active throughout. To this point, there should also be strong evidence of the in-assay mechanistic actions of a given therapy matching its in vivo mechanisms. This might be achieved live in-assay by the test itself, e.g., visualization of a specific immune cell class eliciting MM cell death [44], or could be evidenced as part of test validation, for example, using longitudinal flow cytometry-based profiling to demonstrate the presence and activity of appropriate cell types. In practice, immune system-mediated effects could be enabled by assay design principles that utilize appropriate media formulations and either capture unselected patient BMMCs or incorporate autologous immune cell populations through co-culture, in manners similar to several of the studies reported here.

It would be desirable to work equally effectively with fresh and cryopreserved samples, as well as intra- and extramedullary disease, an ability claimed by several studies herein for the former [41,47,67] and latter [51,72], respectively.

There is the question of whether an ideal test should be 2D, 3D, or dynamic. As previously stated, we believe that dynamic systems hold promise for the future but are unlikely to form the basis of an ideal assay in the near term. Both 2D and 3D assays have shown clinically correlative benefit with moderately sized cohorts [41,42,44,45,46,48,49,51], which potentially aligns with the concept of a liquid disease in a partially solid BMM [39], whereby both environments are biologically relevant and possess the potential to capture clinically relevant cellular responses. Thus, we believe that an ideal assay will incorporate elements of each condition, providing both a 3D scaffold to mimic the in vivo environment and a liquid component to capture cellular behavior within that associated environment. This assay characteristic could be achieved manually or through natural cellular migration of MM cells from a scaffold material into the surrounding liquid medium. Many specific considerations around the makeup of the 3D scaffold and pertinent assay conditions have recently been reviewed elsewhere [40], thus we will avoid re-stating them here.

A final desirable feature not considered elsewhere in this review, but one we believe to be achievable in the short term, has the potential to revolutionize MM treatment: the ability to detect resistant subclonal cell populations within a sample that shows some level of therapeutic response. Rather than calling such a patient sample responsive, the ability to discriminate between responsive and non-responsive MM cells in an assay presents the possibility of treating with initial and next-line therapies within a single assay, enabling further combination treatment formulation or indicating a potential next-line therapy prior to the near-inevitable next relapse. We believe functionality such as this is likely achievable with the current generation of technologies, assuming the correct combination of technology and assay design, and offers great potential, particularly in MM, where the likelihood of relapse is so high.

### 5.5. The Path to Clinical Adoption

Of the assays described in this review article, only EMMA and the assay of Coffey et al. [51] have achieved CLIA approval, and My-DST is understood to be in the process. While both EMMA and My-DST have reported use in clinical trials, only Coffey et al. [51] have reported assay use in a prospective clinical trial. The only ongoing clinical trial we know of involving any of these assays is a single-institution phase II clinical trial evaluating My-DST Comb prospectively in RRMM patients, comparing ex vivo drug-sensitivity results with subsequent clinical responses to Selinexor-based combination therapies. An initial report from the study [64] described a clinically diverse cohort of 18 RRMM patients and concluded that Selinexor/dexamethasone-based triplet drug regimens were associated with high response rates and that My-DST testing of 13 patient samples was 90% sensitive and 67% specific for clinical response. The study is ongoing with an expected end date of late 2026.

We are unaware of any ex vivo MM assays that are currently in widespread clinical use. To achieve such adoption, many of the characteristics described above will likely be necessary. Arguably, some of the more mature assays we have described already possess a significant proportion of the ideal characteristics we have discussed. Nonetheless, barriers to broader adoption remain. From a regulatory perspective, broad clinical use currently necessitates testing in a CLIA-certified laboratory, with most assays operating as LDTs with CLIA oversight. However, widespread adoption will quite possibly be contingent on factors that include prospective clinical validation involving collaboration between MM clinicians and assay developers, achieving payer coverage, and attaining inclusion in clinical guidelines. Future changes to current guidelines may necessitate new validation pathways or the approval of the FDA, but this is not currently the case. While regulatory and accreditation requirements will differ internationally, prospective clinical validation, locally appropriate laboratory oversight, and inclusion in clinical practice guidelines are likely to be common prerequisites for widespread clinical adoption.

## 6. Conclusions

Despite a range of mature and promising options, no clinically correlating MM assay or model provides an all-encompassing solution. The field stands at a critical juncture where technological capabilities are advancing rapidly, but clinical validation, standardization, and practical implementation remain significant challenges. Any clinician, member of the pharmaceutical or biotech industry, or researcher in the MM space will need to determine their need for assay maturity before critically considering the wide-ranging assay characteristics outlined in this review and selecting the path that best suits their needs. Core requirements will most certainly include highly patient-specific assay design, incorporating not only plasma cells, but also immune cells and biological components unique to the individual. Speed and automation will be paramount to enable high-throughput applications and minimize human error, ensuring clinical utility. Although potential solutions exist in the form of established assays, commercial sample media, and research platforms, there are significant deficiencies that need to be overcome. Active research and development by ourselves and others, including several of the groups discussed herein, continue in this space, including efforts that build upon the principles described in this review, such as preservation or reconstitution of patient-specific immune components and functional interrogation of immune-mediated tumor cell killing. It is our hope that these efforts will soon converge on an ideal, broadly applicable solution that will enable radical improvement in MM patient care across all phases of the disease.

## Figures and Tables

**Table 1:** (**A**) Summarized characteristics of 2D models and assays. (**B**) Summarized characteristics of 3D models and assays. (**C**) Summarized characteristics of dynamic models and assays.

Study	Year	System Type	Cells Included	Fresh and/or Cryopreserved	System Type	Drugs Tested	Single Drug or Combo	Vessel	Sensitivity Measure	Clinical Correlation	Doses/Replicates	Turnaround Time	Clinically Approved	No Cells?
**(A)**														
EMMA—[48]	2017	2D	Patient CD138+ cells co-cultured with previously established human bone marrow stroma (bone marrow mesenchymal stem cells, BMSC) and collagen and culture media enriched with patient plasma cells	Fresh BMA	Brightfield microscopy + custom imaging and mathematical algorithms	31 standard-of-care and experimental agents tested (with 127 theoretically possible in a larger plate format)	Single	384 wells for 31 drugs (or 1536 wells for 127 drugs)	Sensitivity defined by patient/drug-specific mathematical models	3-month response; 52 patients; 13 NDMM; 39 RRMM; 96% correctly classified by binary response; 79% using IMWG criteria	5 concentrations (1:3 serial dilution) and 2 replicates	~5 days	Yes. CLIA LDT. (>600 samples reported by Renatino-Canevarolo et al., 2023 [55])	4000 MM cells per well (×5 concentrations ×2 replicates ×31 drugs)
My-DST—[41]	2020	2D	Unselected MNCs from patients with MM	Fresh or cryopreserved BMA	Flow cytometry	7 drugs spanning PIs (Bortezomib, Carfilzomib), IMiDs (Lenalidomide, Pomalidomide), corticosteroids (Dexamethasone), alkylating agents (Cyclophosphamide) and MAbs (Daratumumab)	Single-agent testing; combo response inferred using My-DST Comb algorithm (mathematical prediction only)	96-well plates	My-DST Comb cutoff of 50% (mathematically derived product of % killing across all drugs)	55 unselected, fresh BM samples from MM cases spanning first diagnosis (24), first relapse (12), and multiple relapse (19). 30 used in clincorr. Patients experienced statistically worse event-free survival (EFS) if their next regimen did not contain at least two drugs to which they were predicted sensitive ex vivo by My-DST My-DST Comb correlated strongly with the IMWG depth of clinical response after 4 treatment cycles (*p* = 0.0006) With a clinical response cutoff of a 50% decrease in disease (PR or better), based on the change in MM-specific paraprotein using IMWG criteria, My-DST Comb cutoff of 50% was 96% sensitive (22 of 23 true positives) and 88% specific (6 of 7 true negatives) My-DST results compared with depth of subsequent clinical response after 4 treatment cycles	Single pre-optimized concentration and 3 replicates	~48 h	Reportedly in process of CLIA approval	90,000 MNCs per well (×1 concentration ×3 replicates ×7 drugs)
Coffey et al. assay—[51]	2021	2D	CD138-selected mononuclear plasma cells from BMAs or single cell suspensions derived from mechanical dissociation of plasmacytomas	Fresh BMAs or single cell suspensions	CellTiter-Glo luminescent cell viability assay	170 approved or investigational compounds	Single-agent	384 well plates coated with a protein matrix	Patient defined as sensitive if the IC_50_ was ≤0.2 μM, and this was achievable safely in patients per pharmacokinetic data	25 patients with RR MM; Prospective clinical trial; 16 patients with sufficient material for screening; 13 had treatment guided by test; 92% achieved stable disease or better using IMWG criteria Median PFS was 28 days for patients whose mean AUC was in the top 50th percentile of resistance compared with 139 days for those whose mean AUC was in the bottom 50th percentile of responsiveness (log-rank *p* = 0.042). However, there was no significant difference between high and low AUC with respect to OS (log- rank *p* = 0.151)	8-point drug concentration range, replicates not described	~5 days	Yes—CLIA approved assay	500–4000 CD138+ cells per well (×8 concentrations × replicates unknown ×170 drugs)
Giliberto DSS study—[45]	2022	2D	Purified CD138+ MM cells enriched from BM mononuclear cells	Fresh bone marrow-derived samples	CellTiter-Glo luminescent cell viability assay	Approved and investigational agents: 30 single agents, 19 double agents, and 25 triple-agent combinations	Single agents and ex vivo drug combinations (2- or 3-agent)	Drug-coated 384-well TC plates	Dose–response was used to calculate a modified DSS score ranging from 0 to 100 for each drug	A total of 44 samples at first diagnosis or relapse; observational findings of potential clinical relevance. 13 patients (5 NDMM, 8 RMM) treated with double or triple combinations were considered in clinical correlation, ex vivo DSS scores trended higher in clinical responders (*n* = 9) than in poor responders (*n* = 4), though without formal statistical correlation, using IMWG criteria	Single agents tested at 6 concentrations; double combinations used 5 concentrations for one drug + fixed IC20 priming drug; triple combinations used a 4 × 4 matrix for two drugs (0.1–100 nM) + fixed IC20 third drug; replicates not specified	~5 days	No	5000 CD138+ cells per well (×6 concentrations × replicates unknown × 30 drugs for single drug assay only)
**(B)**														
rEnd/rBM/r-Bone model—[50,66,67]	2008–2020	Latest version is 3D bone-marrow-specific ECM scaffold (collagen I + bone proteins such as fibronectin/osteopontin) combined with myeloma-supportive soluble factors	Primary bone marrow mononuclear cells from patient BM aspirates, maintaining MM plasma cells and incorporating cellular (hematopoietic & stromal) and extracellular components (extracellular matrix & secretory factors)	Fresh BMA in paper. zPredicta website states cryopreserved samples can be used	Flow cytometry used in 2020 study	Cells were treated according to the clinical regimen selected by the treating physician in 2020 study	Combinations used in 2020 study	Unknown/variable. 2008 study used 48-well plates	Plasma and non-plasma cell populations were evaluated post treatment and degree of cell death (by flow cytometry) correlated with clinical response	2020 study used 21 cases “with multiple myeloma”. Showed ~90% accuracy (19/21 cases correct) with 8 true responders and 11 true non-responders identified (2 false positives) using IMWG-like criteria (reported in commercial communications)	Unknown/variable	Unknown. 5-day culture pre-dosing and flow cytometry in 2020 study	No	Varies. zPredicta website states “10–80,000 cells per well in a 96-well plate”
Braham et al. BM Model—[47]	2019	3D Matrigel	Patient CD138+ cells co-cultured with human MSCs and EPCs	Cryopreserved primary MM cells from BMA	Flow cytometry and confocal imaging	A panel of 7 drugs (lenalidomide, pomalidomide, thalidomide, bortezomib, carfilzomib, melphalan, 4-hydroperoxy-cyclophosphamide)	Single	3D Matrigel plugs; plate format not reported	% dead and live-cell count used (% dead showed best performance)	7 relapsed/refractory patients. Inn this study, the use of IMWG-defined response criteria is not explicitly stated, and clinical correlation was established using study-specific definitions. High predictive agreement for AAs and PIs (PPV and NPV ranging from 1.00 to 0.80 for strict outcomes and lower for extended ranging from 1.00 to 0.44). No significant killing by IMiDs even at high doses	A single and a double dose of drug at a concentration known to be effective in 2D and 3D culture	14 days culture + 3 days treatment before readout	No	Not reported
Alhallak’s study—[46] based on earlier model described by [70]	2021	3D matrix formed by cross-linking patient BM endogenous fibrinogen supplemented with purified human fibrinogen and collagen	States “all the accessory and primary cancer cells found in the bone marrow (BM), as well as growth factors, enzymes, and cytokines naturally found in the TME”	Fresh BMA	Flow cytometry	Panel of 11 drugs (carfilzomib, bortezomib, ixazomib, panobinostat, lenalidomide, pomalidomide, dexamethasone, etoposide, doxorubicin, daratumumab, and melphalan)	Single, double, or triple combination (ex vivo) depending on patient clinical treatment regimen	96-well plate	Samples defined as responsive based on significant loss of viability (*p* < 0.05 by ANOVA)	19 RR patients. Treated with upcoming clinical regimen. Predictions concurrent with clinical outcome in 89% of cases, correctly identifying 100% of non-responders and 75% of responders Ex vivo response analyzed by ANOVA results provided to clinical team, who determined clinical response after a cycle of respective regimen, defined according to IMWG criteria and correlated ex vivo response with the clinical response	0×, 3× and 10× Css concentrations (based on pharmacokinetic data from phase 1 and/or phase 2 clinical trials) in quadruplicate	“Less than a week”, including culture, 4 days treatment, and readout	No	100,000 BMNCs per well (×3 concentrations including vehicle ×4 replicates ×1 treatment condition per patient)
Jakubikova PuraMatrix™ model—[42]	2016	3D self-assembling PuraMatrix™ hydrogel	Co-cultured primary MM patient BM cells from BMAs with MSCs in the hydrogel	Fresh BMA	Flow cytometry	Panel of 8 drugs in total (2 for clinical correlation work): (pomalidomide. lenalidomide, thalidomide, bortezomib, carfilzomib, doxorubicin, dexamethasone, melphalan)	Single	96-well plate	Sensitivity defined by fold change of PCs relative to control under 2D vs 3D co-culture conditions	52 patients in total. Patient-level correlative data in the study was limited to 4 patients tested for correlation to two drugs (pomalidomide and carfilzomib). Resistance to pomalidomide was observed in two patients, and one patient showed resistance to carfilzomib in the 3D model, but not in the 2D model, with the 3D model better mimicking known clinical course. Compared carfilzomib and pomalidomide responses in 2D versus 3D MSC co-cultures using samples from four patients across multiple myeloma disease stages. Concluded that enhanced resistance in the 3D system mirrored clinical resistance, despite clinical correlation being based on limited patient numbers and non-standardized response assessment	One concentration per drug with no explicit mention of replicates	Clinical correlation samples treated for 7 days post-culture, but no specific turnaround time provided	No	Not reported.
**(C)**														
Ferrarini et al. RCCS™ Bioreactor study—[72]	2013	Dynamic	PCs, CD138+ MM cells, stromal cells, endothelial cells (bone lamellae and vessels arteriolae reported to be maintained)	Fresh. Extramedullary tissue was obtained from two patients. A skull lesion was excised in a bortezomib-sensitive patient. Excised subcutaneous samples were obtained from one bortezomib-refractory patient	Varied. FACS analysis, TEM, histological analysis, IHC	Bortezomib only	Single	RCCS™ Bioreactor	Clinical correlation sensitivity assessed using histological and immunohistochemical examination of explants, focused on the presence or disappearance of plasma cells. Wider investigative work used FACS, TEM, and histological parameters	5 patients total in study. Only 2 patients tested for clinical correlation (one sensitive and one refractory). Study indicated assay response reflective of clinical response in each patient, using histological and immunohistochemical examination of explants, focused on the presence or disappearance of plasma cells. Response assessment was qualitative and not reported using standardized IMWG response criteria	Tested with and without single dose bortezomib. Replicates not reported	2 patient samples cultured for up to seven days with drug before readout	No	Not specified. Assay used intact tissue explants
Pak et al. MicroC(3) study—[49]	2015	Dynamic	CD138+ tumor cells sorted and cultured with the patients’ own CD138 non-tumor mononuclear cell fractions i.e., MicroC(3)	Fresh BMA	Fluorescence microscopy	Bortezomib only	Single	Custom microfluidics system [73]	Sensitivity defined using k-means and Gaussian mixture model unsupervised clustering	17 patients. Mixture of newly diagnosed, relapsed/refractory, relapsed, sensitive, and refractory. 8 with BMA pre-therapy and 9 with BMA post-therapy. All 17 patients were correctly classified using IMWG criteria	Tested with 2 doses of bortezomib and vehicle	3 days	No	7500 CD138+ and 2 × 8000 CD138− cells per drug/dose condition (×3 doses)
Kruger Vivacyte study—[44]	2024	Dynamic	BMMCs	EDTA BM samples processed on the day of collection	Microwell-based fluorescence imaging (the Cellply Vivacyte with CC-Array)	A panel of 6 drugs (bortezomib, melphalan, dexamethasone, lenalidomide, daratumumab, elotuzumab)	Single	CC-Array microfluidic device	≤90% viable tumor cells (≥10% kill) was used to classify responders	22 patients (12 ND, 10 RR); For 8 patients with clinical follow-up, ex vivo bortezomib sensitivity correctly identified all responders and non-responders. For melphalan, 4 of 5 evaluable patients were correctly classified. Dexamethasone sensitivity was observed in all 4 tested patients, aligning with clinical VGPR or better. No explicit statement that clinical outcomes were defined using standardized IMWG response criteria.	Not specified.	No standardized assay time provided. Drug sensitivity analysis was possible over a duration of ~2 days for most patient samples	No	10–20 MNCs per microwell (×1200 microwells per channel/1 channel per condition ×6 drugs. Replicates and concentrations unknown)

## Data Availability

No new data were created or analyzed in this study. Data sharing is not applicable to this article.

## Abbreviations

The following abbreviations are used in this manuscript:

MM	Multiple myeloma
BM	Bone marrow
M-protein	Monoclonal protein
IMWG	International Myeloma Working Group
NDMM	Newly diagnosed multiple myeloma
MRD	Minimal residual disease
ctDNA	Circulating tumor DNA
ASCT	Autologous stem cell transplantation
IMiDs	Immunomodulatory drugs
PIs	Proteasome inhibitors
MAbs	Monoclonal antibodies
BSAbs	Bispecific antibodies
ADCs	Antibody–drug conjugates
CAR-T	Chimeric antigen receptor T-cell
RRMM	Relapsed of refractory multiple myeloma
PFS	Progression-free survival
LOT	Lines of therapy
BMM	Bone marrow microenvironment
EMMA	Ex vivo Mathematical Myeloma Advisor
BMA	Bone marrow aspirate
LDT	Laboratory-developed test
CLIA	Clinical Laboratory Improvement Amendments
My-DST	Myeloma drug sensitivity testing
EFS	Event-free survival
HTS	High-throughput screening
CTG	CellTiter-Glo
IC50	Half maximal inhibitory concentration
AUC	Area under the curve
OS	Overall survival
BMMCs	Bone marrow mononuclear cells
TC	Tissue culture
DSS	Drug sensitivity score
CR	Complete response
VGPR	Very good partial response
SD	Stable disease
PD	Progressive disease
rEnd	Reconstructed endosteal
rBM	Reconstructed bone marrow
MSCs	Mesenchymal stem cells
EPCs	Endothelial progenitor cells
AA	Alkylating agent
PPV	Positive predictive value
NPV	Negative predictive value
3DTEBM**^®^**	3D tissue-engineered bone marrow
Css	Steady-state plasma drug concentration
PR	Partial response
ECM	Extracellular matrix
MNCs	Mononuclear cells
TEM	Transmission electron microscopy
MicroMC	Mono-co-culture system
MicroC(3)	Cis-co-culture system
R-ISS	Revised Multiple Myeloma International Staging System
BiTEs	Bispecific T-cell engagers
EHA	European Hematology Association
NAM	New approach methodology
ND	Newly diagnosed
RR	Relapsed/refractory
